# Clinical and biochemical manifestation of acute viral hepatitis B in Republic of Moldova

**DOI:** 10.1186/1471-2334-14-S7-P14

**Published:** 2014-10-15

**Authors:** Lilia Cojuhari, Victor Pântea, Gheorghe Plăcintă, Valentin Cebotarescu, Olga Chirita, Ana Crudu, Liviu Iarovoi, Zinaida Ieseanu, Cristina Marandici

**Affiliations:** 1 Faculty for Continuing Medical Education, Nicolae Testemițanu State Medical and Pharmacy University, Chişinău, Republic of Moldova; 2 Department of Infectious Diseases, Parasitology and Tropical Medicine, Nicolae Testemițanu State Medical and Pharmacy University, Chişinău, Republic of Moldova

## Background

Hepatitis B viral represents a pathology with a severe impact on public health. Worldwide, approximately 350 million individuals are chronically infected with hepatitis B virus (HBV). HBV is the leading cause of cirrhosis globally. Once chronic infection is established, approximately 30% of the patients will develop cirrhosis, and approximately one-quarter of patients with cirrhosis will develop decompensated liver disease within 5 years. Cirrhosis also substantially increases the risk for hepatocellular carcinoma (HCC).

## Methods

In the study were included 73 patients with acute hepatitis B viral with a mean age of 33.20±1.46 years that were hospitalized in the Toma Ciorbă Infectious Diseases Clinical Hospital. The analysis parameters: age, sex, onset, clinical manifestation, liver size, disease level, total bilirubin, ALAT, thymol test, prothrombin.

## Results

Acute viral hepatitis B has been observed in the both sexes: women – 27 (37%), men – 46 (63%). The disease with the acute onset has been manifested in 73 patients (100%), more frequently in icteric form in 65 patients (89%), than in anicteric form in 8 patients (11%). In 1.4% of acute HBV patients it occurred in a milder form: in 65.8% – mild and in 32.8% – severe form. The preicteric period lasted 7.51±0.57 days. Acute B viral hepatitis includes asthenic, dyspeptic and mesenchymal inflammatory syndrome. Biochemical investigations: increased level of bilirubin 184.54±14.18 mkmol/L, ALT constitutes 11.02±0.38 mmol/h/L, thymol test 11.29±0.74 U and prothrombin index – 70.99±1.51%. Hepatomegaly was 3.5±0.16 cm in all patients (100%), and splenomegaly – 2.0±0.2 cm in 39 patients (53.4%).

## Conclusion

Acute hepatitis B virus affects both sexes, being more frequent in men, and is manifested through acute onset in the icteric form, the moderate form being characterized clinically by the dyspeptic, asthenic, and biochemical syndrome through the ALT activity increase, bilirubin and thymol test.

